# Gene expression analysis of pig cumulus-oocyte complexes stimulated in vitro with follicle stimulating hormone or epidermal growth factor-like peptides

**DOI:** 10.1186/s12958-015-0112-2

**Published:** 2015-10-06

**Authors:** Milan Blaha, Lucie Nemcova, Katerina Vodickova Kepkova, Petr Vodicka, Radek Prochazka

**Affiliations:** Laboratory of Developmental Biology, Institute of Animal Physiology and Genetics, The Czech Academy of Sciences, Rumburska 89, 277 21 Libechov, Czech Republic; Department of Neurology, Massachusetts General Hospital and Harvard Medical School, Charlestown, MA USA

**Keywords:** FSH, Growth factors, Cumulus cell, Transcriptome

## Abstract

**Background:**

The gonadotropin-induced resumption of oocyte meiosis in preovulatory follicles is preceded by expression of epidermal growth factor (EGF)-like peptides, amphiregulin (AREG) and epiregulin (EREG), in mural granulosa and cumulus cells. Both the gonadotropins and the EGF-like peptides possess the capacity to stimulate resumption of oocyte meiosis in vitro via activation of a broad signaling network in cumulus cells. To better understand the rapid genomic actions of gonadotropins (FSH) and EGF-like peptides, we analyzed transcriptomes of cumulus cells at 3 h after their stimulation.

**Methods:**

We hybridized aRNA from cumulus cells to a pig oligonucleotide microarray and compared the transcriptomes of FSH- and AREG/EREG-stimulated cumulus cells with untreated control cells and vice versa. The identified over- and underexpressed genes were subjected to functional genomic analysis according to their molecular and cellular functions. The expression pattern of 50 selected genes with a known or potential function in ovarian development was verified by real-time qRT-PCR.

**Results:**

Both FSH and AREG/EREG increased the expression of genes associated with regulation of cell proliferation, cell migration, blood coagulation and extracellular matrix remodeling. FSH alone induced the expression of genes involved in inflammatory response and in the response to reactive oxygen species. Moreover, FSH stimulated the expression of genes closely related to some ovulatory events either exclusively or significantly more than AREG/EREG (*AREG*, *ADAMTS1*, *HAS2*, *TNFAIP6*, *PLAUR*, *PLAT*, and *HSD17B7*). In contrast to AREG/EREG, FSH also increased the expression of genes coding for key transcription factors (*CEBPB*, *FOS*, *ID1*/*3*, and *NR5A2*), which may contribute to the differing expression profiles of FSH- and AREG/EREG-treated cumulus cells.

**Conclusions:**

The impact of FSH on cumulus cell gene transcription was higher than the impact of EGF-like factors in terms of the number of cell functions affected as well as the number of over- and underexpressed genes. Both FSH and EGF-like factors overexpressed genes involved in the post-ovulatory switch in steroidogenesis and tissue remodelling. However, FSH was remarkably more efficient in the up-regulation of several specific genes essential for ovulation of matured oocytes and also genes that been reported to play an important role in maturation of cumulus-enclosed oocytes in vitro.

**Electronic supplementary material:**

The online version of this article (doi:10.1186/s12958-015-0112-2) contains supplementary material, which is available to authorized users.

## Background

The development of mammalian female germ cells requires close contact and metabolic cooperation with the somatic granulosa cells. In the antral follicles, the granulosa cells differentiate into two phenotypically distinct populations: the mural granulosa cells, lining the follicle wall and the cumulus cells, surrounding the oocyte in several layers. Both populations of granulosa cells fulfil different roles during follicle development: the mural granulosa cells are predominantly involved in the perception of signals from outside follicle, the production of steroid hormones and follicular rupture; the cumulus cells provide nutrients and regulatory molecules for oocyte growth, final maturation and ovulation. In turn, the range of function of cumulus cells, including steroidogenesis, gene expression, extracellular matrix formation and metabolism, are modified by factors secreted by the oocyte [1].

During the growth phase, the oocyte is arrested in the first meiotic prophase, but it gains full meiotic competence in several steps [2]. However, these meiotically competent oocytes still remain in the dictyate stage if they are retained within follicles. They only resume meiosis following the preovulatory surge of luteinizing hormone (LH). The resumption of meiosis is regulated inside oocytes by a post-translation mechanism and does not depend on the activation of genes in the oocyte itself [3]. Nevertheless, the LH-induced meiotic resumption is accompanied by a dramatic change in gene expression profiles in mural granulosa and cumulus cells. The changes in the cumulus/granulosa cell transcriptome are under the control of a broad signaling network activated in follicular cells by the LH surge. This network includes major cellular protein kinases including protein kinase A (PKA), phosphoinositide-3-kinase/v-akt murine thymoma viral oncogene homolog (PI3K/AKT), mitogen-activated kinase 3/1 (MAPK3/1) and MAPK14 [4–6] and downstream transcription factors including CREB, AP1, NRIP1, NR5A2 and CEBPB [7–11].

The resumption of meiosis is accompanied by an expansion of cumulus cells, a process that enables the detachment of the oocyte-cumulus complex from the follicle wall and its ovulation to the oviduct. In response to the preovulatory surge of LH, the cumulus cells start the synthesis of a large amount of hyaluronic acid (HA)-enriched extracellular matrix that is deposited into the extracellular spaces and causes the process of expansion [12]. The production of HA is controlled by hyaluronan synthase 2 (HAS2), which is highly expressed in cumulus cells shortly after the preovulatory surge of LH [13]. The retention and organization of HA in the extracellular matrix is mediated by HA-binding proteins, which include versican [14], tumor necrosis factor α-stimulated gene 6 [15], pentraxin 3 [16] and serum-derived members of the inter-α-trypsin inhibitor (IαI) family [17, 18]. The experiment with mice null for the *Ptgs2* and *Ptger2* gene revealed that the local production of prostaglandin E2 is also essential for the normal expansion of mouse cumulus [19, 20].

Mechanisms of meiotic resumption and cumulus expansion have been studied on a whole follicle culture that is often used in mice and rats or on a simplified model based on a culture of isolated COCs. In the latter case, LH and hCG are inefficient at triggering these events due to the absence of functional LH-receptors on cumulus cells [21, 22]. Functional LH receptors, however, can develop over the course of in vitro culture, under the influence of FSH [22, 23]. For this reason, both the resumption of meiosis and cumulus expansion is routinely stimulated by the addition of FSH, or a combination of FSH and hCG.

The mechanism of LH signal transduction in the preovulatory follicle has been intensively studied over the last decade. It was found in the model of whole follicle cultures in mice and rats that LH binds to its receptors on mural granulosa cells and stimulates the expression of epidermal growth factor (EGF)-like peptides (amphiregulin-AREG, epiregulin-EREG, betacellulin-BTC) [24, 25]. These peptides then act directly on mural granulosa as well as cumulus cells, bind to the EGF-receptor and consequently stimulate the resumption of meiosis and expansion of the cumulus. In addition, a regulatory loop between the mural granulosa and the cumulus cells also ensures the production of AREG and EREG in the cumulus cell compartment [26]. The EGF-like peptides are also produced in the cumulus cells of in vitro-cultured COCs upon stimulation with FSH and trigger the resumption of meiosis [27].

This knowledge offers the possibility of stimulating the meiotic resumption and cumulus expansion of cultured COCs by purified EGF-like peptides. We have recently shown that AREG and EREG stimulate the expansion of cumulus cells, maturation of the oocyte and acquisition of oocyte developmental competence in vitro in pigs [28]. However, we have also pointed out the lower efficiency of EGF-like peptides, compared to FSH, in inducing the expression of expansion-related genes and their inability to activate PKA in cultured COCs [28]. A detailed study of signaling pathways activated by FSH and EGF-like factors in pig cumulus cells confirmed that FSH-stimulated, but not AREG-stimulated resumption of meiosis depends on PKA activity [6]. In addition, this study documented that both modes of stimulation result in the activation of the EGF-receptor tyrosine kinase and MAPK3/1 pathways in cumulus cells, which is essential for both meiotic resumption and cumulus expansion [6]. It is not known how the differences between FSH and AREG/EREG in the activation of signaling pathways affect the gene expression profiles of treated COCs.

We explored the use of microarray hybridization technology to identify rapid global changes in the gene expression profile of cumulus cells stimulated with FSH or EGF-like peptides (AREG/EREG). Both these treatments have been shown to stimulate oocyte meiotic resumption, cumulus cell expansion and to modify cumulus cell steroidogenesis, but it is not completely clear what other molecular and cellular processes in cumulus cells are affected by FSH and EGF-like peptides and what is their relationship to the acquisition of oocyte developmental competence. The current IVM techniques yield oocytes with low developmental competence as compared to their counterparts matured in vivo. The molecular mechanisms underlying this insufficiency are largely unknown. Nevertheless, recent data indicate that the way, by which the maturation of oocytes is stimulated, is crucial for determining the oocyte developmental competence [29–33]. The aim of this study was to assess the differences in impact of FSH and EGF-like peptides on gene expression in cumulus cell with special emphasis on the genes with known relationship to regulation of reproductive function in mammals.

## Methods

The study was conducted in the laboratories of IAPG in Libechov in 2012–2015.

### Collection of cumulus-oocyte complexes

Ovaries were obtained from premature crossbred gilts (Landrace and Large White), 6–8 months old and 90–120 kg in weight, slaughtered at a local abattoir for commercial purposes. The ovaries were excised and transported to the laboratory in a thermo-flask at 38 °C. The contents of medium-size antral follicles about 3–5 mm in diameter were aspirated with a syringe connected to 20 G needle, pooled in a test-tube and allowed to sediment for 10 min. The sediment was washed twice with PBS, placed in a Petri dish and the COCs were collected by a pipette. Only COCs surrounded by compact multi-layered cumulus were selected for experiments.

### Culture of cumulus-oocyte complexes in vitro

The COCs were cultured in M-199 medium (Gibco, Life Technologies, Rockville, USA) supplemented with 0.91 mM sodium pyruvate, 0.57 mM cysteine, 5.5 mM Hepes, antibiotics and fetal calf serum (5 %) (all Sigma, Prague, Czech Republic). Groups of 25–30 COCs were cultured in four-well dishes (Nunclon, Roskilde, Denmark) in 0.5 ml of culture medium at 38.5 °C in a humidified atmosphere of 5 % CO_2_ for 3 h. The maturation of COCs was stimulated by the addition of human recombinant FSH (100 ng/ml; Gonal F; Merck Serono Europe, London, GB) or recombinant human AREG (100 ng/ml, Sigma) and EREG (100 ng/ml; R&D Systems, Minneapolis, MN, USA) to the culture medium. These concentrations proved to activate various signaling pathways in cumulus cells, stimulate cumulus expansion and oocyte maturation in our previous studies [6, 28]. At the end of the culture period, about 150 COCs were transferred to a Petri dish with 2 ml of PBS and the cumulus cells were mechanically stripped of oocytes by pipetting. The oocytes were removed and PBS with a suspension of the separated cumulus cells was transferred to a microtube and briefly centrifuged in a microcentrifuge. The supernatant was discarded and the pellet of cumulus cells was lysed in 300 μl of RTL buffer (Qiagen, Hilden, Germany) and stored at −80 °C. In each experiment, groups of control, FSH- or AREG/EREG-stimulated COCs were cultured for 42 h as a biological control for the microarray. These COCs were assessed for cumulus expansion and oocyte maturation by methods described in our previous papers [6, 28]. The cumulus expansion index was 0; 2.98 ± 0.05; and 2.74 ± 0.04 and the maturation rate of oocytes to metaphase II was 17.5 ± 1.4; 93.2 ± 3.8; 73.6 ± 2.9 for control, FSH and AREG/EREG group, respectively.

### Microarray

**Fig. 1:**
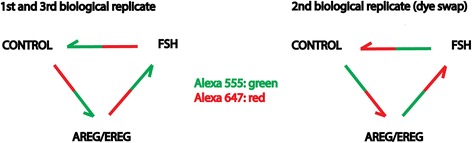
Design of hybridization scheme of microarray experiment. Each *arrow* represents one microarray. Samples of cumulus cells stimulated with AREG/EREG, FSH or control cells were labeled with AlexaFluor 555 (depicted in *green*) or AlexaFluor 647 (depicted in *red*)

*Experimental design of microarray experiment*. Three different samples (FSH treated, AREG/EREG treated and control untreated cumulus cells), each represented by 3 independently prepared biological replicates, were hybridized to 9 microarrays (Pigoligoarray, www.pigoligoarray.org) as follows (Fig. 1):

1st and 3rd biological replicate: Control AlexaFluor 647+ AREG/EREG AlexaFluor 555; AREG/EREG AlexaFluor 647 + FSH AlexaFluor 555; FSH AlexaFluor 647 + Control AlexaFluor 555. 2nd biological replicate (dye swap): Control AlexaFluor 555 + AREG /EREG AlexaFluor 647; AREG/EREG AlexaFluor 555 + FSH AlexaFluor 647; FSH AlexaFluor 555 + Control AlexaFluor 647.

*Total RNA isolation*. Total RNA from all nine samples was isolated using an RNeasy PLUS mini kit (Qiagen) according to the manufacturer’s instructions. RNA integrity was verified using an RNA 6000 Nano LabChip kit with an Agilent 2100 Bioanalyzer. Only RNA samples with a resulting RNA integrity number (RIN) between 8.2 and 9.1 were used for the amplification of aRNA.

*aRNA amplification and labeling*. aRNA was prepared with one-round amplification using an AminoAllyl MessageAmp II Kit (Ambion, Austin, Texas, USA). The in vitro transcription time was 14 h. Five microgram of aRNA were conjugated with either Alexa Fluor 555 or 647 dye (Invitrogen, Carlsbad, California, USA). Labeled aRNAs were purified using a PicoPure RNA isolation kit (Arcturus) according to the manufacturer’s instructions; 2 μg of each labeled aRNA (Alexa Fluor 555- and Alexa Fluor 647-conjugated aRNA samples according to the experimental design) were mixed together and stored at −80 °C.

*Hydration and crosslink of microarrays*. Microarray slides were rehydrated for 10 s above a water bath at 50 °C, dried on a heat block for 5 s at 65 °C and cooled for 1 min at room temperature. This procedure was repeated 4 times. UV cross-linking was performed by exposing rehydrated slides to 180 mJ of UV radiation. After crosslinking, the slides were washed in 0.1 % SDS with constant gentle mixing for 5 min followed by rinsing in ddH_2_O and 3 min of incubation in 100 % ethanol with constant gentle mixing, all at room temperature. Slides were dried by centrifugation for 4 min at 200 g. Just before use, the microarray slides were pre-hybridized at 42 °C for 1 h in pre-hybridization buffer (5 × SSC, 0.1 % SDS, 1 % BSA) using a Tecan HS400Pro hybridization station (TECAN, Austria).

*Probe fragmentation and hybridization*. Pre-mixed labeled aRNA probes were fragmented using Fragmentation buffer (Ambion Austin, Texas, USA) according to the manufacturer’s instructions. After fragmentation, probes were denatured for 5 min at 90 °C, mixed with 130 μl of SlideHyb Glass Array Hybridization Buffer # 1 (Ambion Austin, Texas, USA) preheated to 68 °C and hybridized for 15 h at 42 °C in Tecan HS400Pro to pre-hybridized microarray slides. After hybridization, slides were washed and dried automatically in the Tecan HS400Pro instrument and stored in the dark until scanned the same day.

*Scanning and image analysis*. Slides were scanned using a High-Resolution Microarray Scanner (Agilent) at 5 μm resolution with the 20-bit dynamic range setting. Agilent Feature Extraction Software (v10.7) was used for image analysis.

*Microarray data analysis*. Microarray data background correction, normalization and statistical inference of changes in gene expression were done using the Bioconductor package Limma in the R statistical environment. Briefly, spot and background median signals were imported into the R data-frame and normex background correction was applied. After print-tip loess within-array normalization, A-quantile normalization was used to make the signal intensities more comparable across arrays. A linear model was fitted, with coefficients estimated for all 3 possible comparisons (FSH vs Control, AREG/EREG vs Control and FSH vs AREG/EREG) and for a possible dye-effect. The empirical Bayes method to moderate the standard errors of the estimated changes in expression was applied as implemented in Limma. *P*-values were adjusted according to the Benjamini and Hochberg method to control for false discovery rate. Only genes with adjusted *p*-value <0.01 were considered significantly changed.

The functional role of the differentially expressed genes was assessed for gene ontology using the freely available tool Database for Annotation, Visualization and Integrated Discovery (DAVID) (http://david.abcc.ncifcrf.gov/).

### Quantitative real-time PCR

To validate the microarray data, 50 candidate genes were selected for further analysis by real-time PCR. For this, sequence specific primers (Additional file 1: Table S1) were designed using Beacon Designer (Premier Biosoft, CA, USA). The total RNA was isolated from cumulus cells of 30 COCs cultured for 3 h using an RNeasy Mini Kit (Qiagen, Hilden, Germany) following the manufacturer’s instructions. Real-time PCR was carried out in a RotorGene 3000 cycler (Corbett Research, Sydney, Australia) using a One-Step RT-PCR Kit (Qiagen) with gene-specific primers. The 20 μl total reaction volume contained QIAGEN OneStep RT-PCR Buffer (1×), dNTP Mix (400 μM final concentration of each), reverse and forward primers (both 400 nM final concentration), SybrGreenI (0.4 μl of 1:1000 stock solution, Molecular Probes, Eugene, OR, USA), RNasine inhibitor (5 IU, Promega, Madison, WI, USA), QIAGEN OneStep RT-PCR Enzyme Mix (0.8 μl), and template RNA (2 μl). The reaction conditions were as follows: reverse transcription at 50 °C for 30 min, pre-denaturation at 95 °C for 15 min, followed by various numbers of PCR cycles, each of which consisted of denaturation at 95 °C for 30 s, annealing at temperature specific for each pair of primers (shown in Additional file 1: Table S1) for 20 s, extension at 72 °C for 20 s, and a final extension step at 72 °C for 5 min. The specificity of PCR product was verified by melting analysis. The relative concentrations of the templates in the different samples were determined using comparative analysis software (Corbett Research). The results for individual target genes were normalized according to the relative concentration of the internal standard, *HPRT*.

## Results

### Microarray analysis

We identified 2981 transcripts as overexpressed and 3582 as underexpressed in cumulus cells stimulated with FSH for 3 h compared to control unstimulated cells. A comparison of AREG/EREG-stimulated cells with control cells revealed 1491 transcripts as overexpressed and 1783 transcripts as underexpressed. Altogether, 2899 transcripts were overexpressed and 2581 transcripts underexpressed in the AREG/EREG group compared to the FSH group. The complete microarray data have been deposited in GEO and are accessible via the link http://www.ncbi.nlm.nih.gov/geo/ (GEO Accession: GSE64858). From the identified significantly changed transcripts (*p* < 0.01), only those transcripts with a log2 fold-change above 1.5 were used in downstream analyses.

**Fig. 2:**
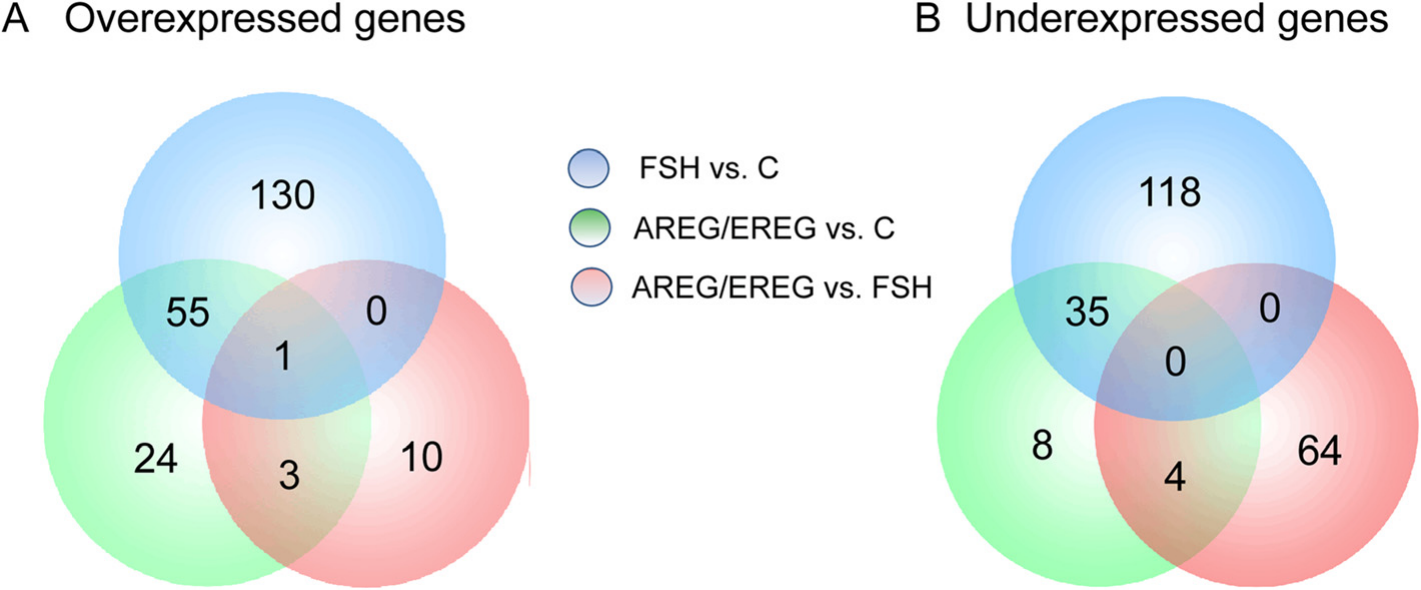
Venn diagrams summarizing microarray data analysis. **a** Venn diagram showing numbers of overexpressed and (**b**) underexpressed genes in cumulus cells after stimulation of COCs with FSH or AREG/EREG. Only genes with log2 fold-change above 1.5 were considered for this analysis

The Venn diagrams were constructed to identify the exclusively over- and underexpressed genes after stimulation with AREG/EREG or FSH. As shown in Fig. 2a, we found that 27 genes were exclusively expressed in COCs cultured with EGF-like factors, the expression levels of 55 genes were increased by both AREG/EREG and FSH and 130 transcripts were found to be overexpressed in COCs cultured with FSH alone. A comparison of AREG/EREG-stimulated cells with FSH-stimulated cells revealed 10 overexpressed transcripts. The culture with AREG/EREG decreased the expression levels of 12 genes, 35 transcripts were underexpressed in both AREG/EREG-stimulated and FSH-stimulated COCs, and the expression of 118 genes was only decreased by FSH. A comparison of AREG/EREG-stimulated cells with FSH-stimulated cells revealed 68 underexpressed genes (Fig. 2b).

The analysis of the data revealed that several genes with a well-recognized function in the regulation of oocyte meiotic resumption and cumulus expansion were only overexpressed by FSH, among them genes associated with signal transduction (*AREG*), extracellular matrix synthesis and organization (*ADAMTS1*, *HAS2*, *TNFAIP6*, *PLAT*, and *PLAUR*) and the regulation of transcription (*CEBPB*, *CREM*, *FOXO3*, and *NR4A1*). Both AREG/EREG and FSH induced the expression of *CYP11A1* and *PGR* involved in regulation of progesterone synthesis and signaling. Surprisingly, culture with FSH decreased the expression of genes involved in signal transduction (*IRS1* and *GRB14*) and the regulation of transcription (*PPARG* and *SMAD2*). Moreover, other genes involved in transcriptional regulation (*FOS*, *FOXO1*, *JUN*, and *PNRC1*) were underexpressed under both culture conditions. A complete list of exclusively and commonly over/underexpressed genes after FSH and AREG/EREG stimulation is shown in Additional file 2: Table S2.

### Gene ontology

**Fig. 3:**
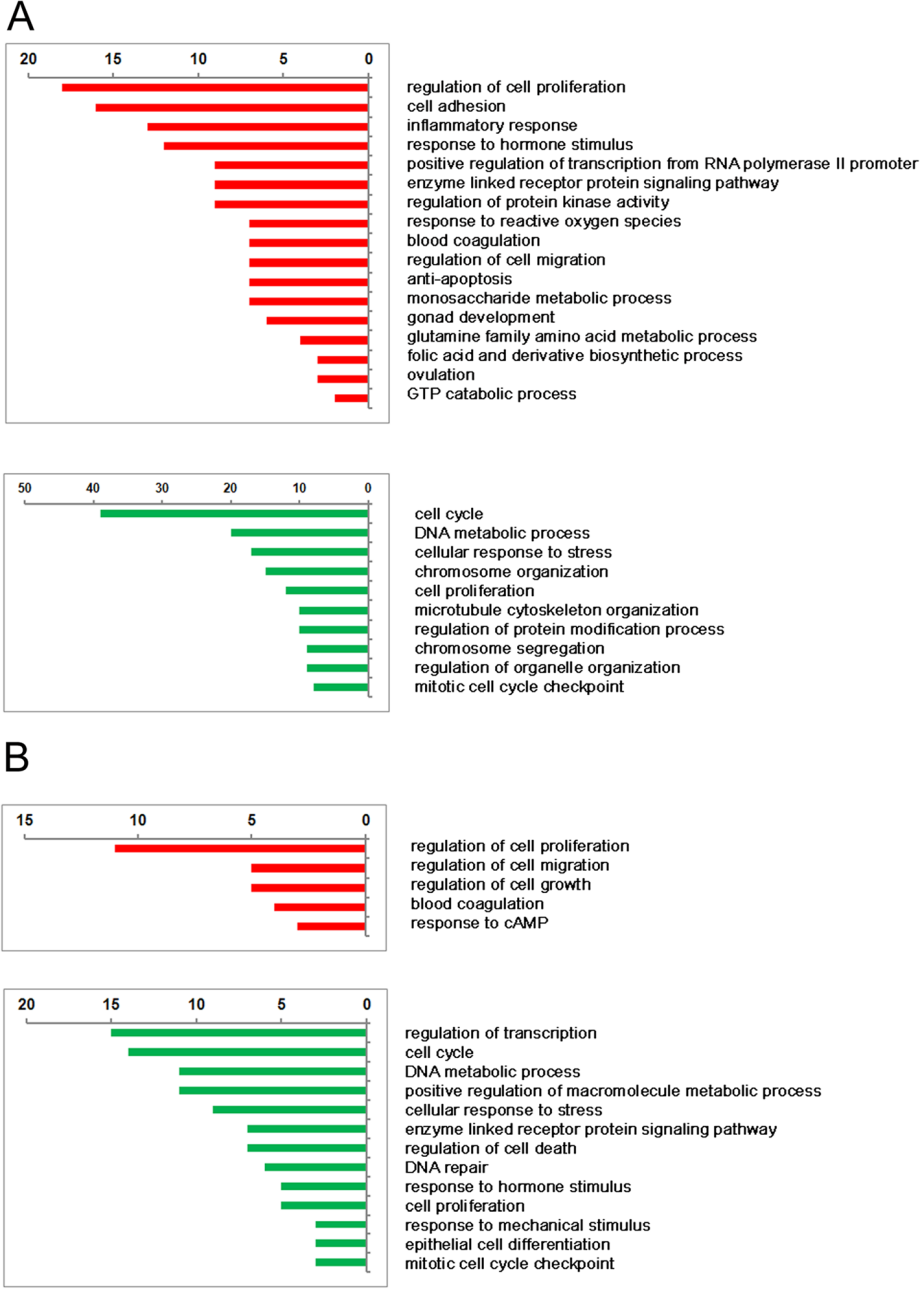
Functional classification and numbers of genes over- and underexpressed in cumulus cells after stimulation with FSH (**a**) or AREG/EREG (**b**). Only genes with log2 fold-change above 1.5 were considered for this analysis. The numbers of genes affected by the treatments are shown at the *top* of the panels. The gene ontology analysis was performed using DAVID Bioinformatic Resources (http://david.abcc.ncifcrf.gov/)

The functional role of the differentially expressed genes was assessed for gene ontology using the freely available tool Database for Annotation, Visualization and Integrated Discovery (DAVID) (http://david.abcc.ncifcrf.gov/). To increase the predictive ability, only terms with *p* values ≤0.05 were considered. The selected enriched biological processes are shown in Fig. 3. In general, treatment with FSH affected the expression of approx. double the number of genes with log2 > 1.5 than treatment with AREG/EREG. Both FSH and AREG/EREG stimulated the expression of genes associated with the regulation of cell proliferation and cell migration. Interestingly, both culture conditions increased the expression of genes involved in blood coagulation and extracellular matrix remodeling (*F3*, *FBLN5*, *TFPI2*, and *SERPINE1*). In addition to AREG/EREG, FSH induced the expression of tissue plasminogen activator (*PLAT*) and urokinase plasminogen activator receptor (PLAUR). FSH also upregulated a wide variety of genes associated with inflammatory response (*ANXA1*, *BMPR1B*, *CCL4L1*, *CEBPB*, *F3TAC1*, *F11R*, *LTA4H*, *OLR1*, *PRDX2*, *TGFB1*, and *TNFAIP6*) and with the response to reactive oxygen species (*CYP11A1*, *CRYAB*, *MT3*, *OLR1*, *PRDX2*, *SDC1*, and *SERPINE1*). As expected, the cultures with FSH increased the expression of genes involved in gonad development and ovulation (*ADAMTS1*, *BMPR1B*, *FGF9*, *FOXO3*, *PGR*, and *SDC1*). The genes involved in the cell cycle, DNA metabolism, response to stress, cell proliferation and mitotic cell cycle checkpoint were underexpressed under both culture conditions. In the list of transcripts underexpressed in COCs cultured with AREG/EREG, the analysis revealed a group of genes (*ADM*, *FOXO1*, *FOS*, *GCLC*, and *TXNIP*) associated with the response to hormonal stimuli (Fig. 3).

### Real-time RT-PCR validation

**Fig. 4:**
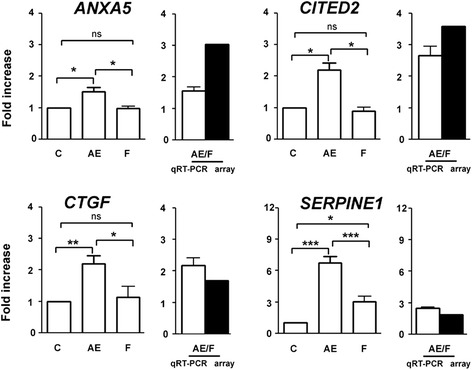
Quantitative real-time PCR validation of genes found to be overexpressed in AREG/EREG vs. FSH microarray. The relative abundance of specific gene mRNA is expressed in arbitrary units as fold increases in the specific gene /*HPRT* ratio over the level found in the control group at the time 0 h. *C* control, *AE* AREG/EREG, *F* FSH. *P* < 0.05 for *single asterisk*; *P* < 0.01 for *double asterisk*; *P* < 0.001 for *triple asterisk*

**Fig. 5:**
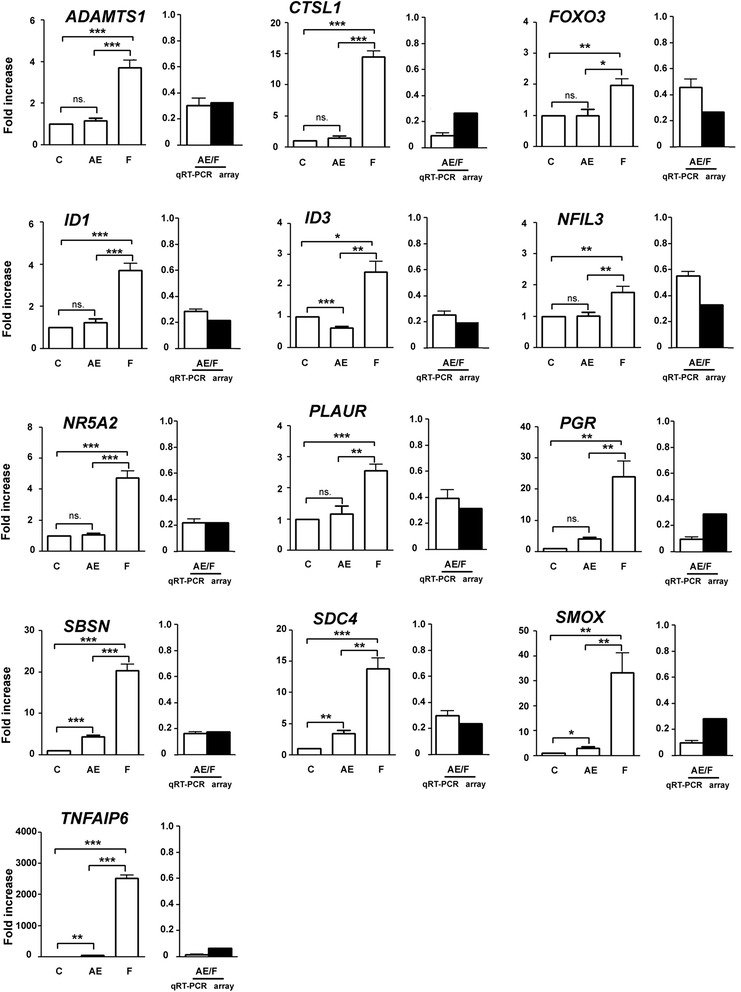
Quantitative real-time PCR validation of genes found to be underexpressed in AREG/EREG vs. FSH microarray. The relative abundance of specific gene mRNA is expressed in arbitrary units as fold increases in the specific gene /*HPRT* ratio over the level found in the control group at the time 0 h. *C* control, *AE* AREG/EREG, *F* FSH. *P* < 0.05 for *single asterisk*; *P* < 0.01 for *double asterisk*; *P* < 0.001 for *triple asterisk*

To confirm the microarray data analysis, we quantified the expression levels of 50 selected genes by quantitative real-time PCR (qRT-PCR). The candidate genes selected were those that had previously been identified as being involved in the physiology of reproduction. Table 1 shows the results of validation for the FSH vs. control and AREG/EREG vs. control microarray. The validation confirmed a significant increase in expression for 21 transcripts (78 %) selected from the list of overexpressed genes originating from the FSH vs. control microarray. The list of confirmed transcripts includes factors essential for cumulus expansion (*HAS2* and *TNFAIP6*), follicle tissue remodeling (*ADAMTS1*), gonadotropin-dependent EGFR signaling (*AREG*), polyamine metabolism (*ODC1* and *SMOX*), plasminogen metabolism (*PLAT*, *PLAUR*, and *SERPINE1*) and transcription factors (*CEBPB* and *PGR*). However, only 4 transcripts (33 %; *FST*, *PPARG*, *PTGES* and *RGS3*) selected from the list of underexpressed genes were found to be significantly decreased, as measured by qRT-PCR. Similar results were obtained with the AREG/EREG vs. control microarray. The expression pattern was confirmed for 9 of 12 selected genes overexpressed in the microarray (75 %) and only 3 genes (27 %; *JUN*, *PNRC1*, and *RGS3*) selected from underexpressed ones were confirmed by qRT-PCR. With the AREG/EREG vs. FSH array, qRT-PCR confirmed the microarray results for all transcripts selected from the lists of overexpressed (4) and underexpressed (13) genes (Figs. 4 and 5). Interestingly, qRT-PCR revealed significantly different expression patterns of key transcription factors (*FOXO3*, *ID1*, *ID3*, *NFIL3*, *NR5A2*, and *PGR*) between FSH- and AREG/EREG-stimulated cells. Additionally, the expression levels of *CTSL*, *PGR*, *SBSN*, *SDC4*, *SMOX* and *TNFAIP6* were increased dramatically in cells cultured with FSH in comparison with AREG/EREG-treated cells.

**Table 1:** Verification of selected differentially expressed genes by real time RT-PCR

Probe name	Gene symbol	Gene description	Fold change in gene expression values determined by RT-PCR (Mean ± SEM)	Significance vs. non-stimulated control	Array results (Fold change)
	FSH vs. Control - overexpressed				
31605:45360_15417313:f	*ABCC5*	ATP-binding cassette, sub-family C (CFTR/MRP), member 5	0.83 ± 0.12	ns.	4.79
9436:30168_CL9Contig1:f	*ADAMTS1*	ADAM metallopeptidase with thrombospondin type 1 motif, 1	3.72 ± 0.36	***	6.95
13062:45360_CL23Contig1:f	*ANXA1*	Annexun A1	0.93 ± 0.10	ns.	3.15
1117:45360_CL10Contig1:r	*ANXA2*	Annexin A2	0.98 ± 0.07	ns.	4.02
14127:9388_21549088:r	*AREG*	Amphiregulin	5.04 ± 0.46	***	4.06
18133:7423_CL1Contig2:r	*CEBPB*	CCAAT/enhancer binding protein (C/EBP), beta	9.68 ± 1.58	***	2.89
6532:35657_CL1Contig1:r	*CYP11A1*	Cytochrome P450, family 11, subfamily A, polypeptide 1	11.92 ± 0.90	***	16.20
TC226972:f	*FOXO3*	Forkhead box O3	1.97 ± 0.21	**	4.22
TC235800:f	*HAS2*	Hyaluronan synthase 2	47.63 ± 5.11	***	6.59
9355:35407_CL1Contig1:f	*HSD17B7*	Hydroxysteroid (17-beta) dehydrogenase 7	3.35 ± 0.30	***	3.87
5795:24780_CL1Contig1:r	*MARCKSL1*	MARCKS-like 1	4.44 ± 0.53	***	2.85
1337:12192_CL1Contig2:f	*ODC1*	Ornithine decarboxylase 1	6.22 ± 0.85	**	5.26
15979:340_CL1Contig1:f	*PBX1*	Pre-B-cell leukemia homeobox 1	0.70 ± 0.10	*	4.24
31453:2453_233792:f	*PGR*	Progesterone receptor	23.99 ± 5.10	**	15.57
8538:3878_CL2Contig1:f	*PLAT*	Plasminogen activator, tissue	14.16 ± 2.36	***	5.48
4143:1539_CL1Contig1:r	*PLAUR*	Plasminogen activator, urokinase receptor	2.56 ± 0.20	***	4.00
33691:1766_29276590:r	*PRDX2*	Peroxiredoxin 2	6.30 ± 0.44	***	2.88
13439:18092_CL1Contig1:r	*RBKS*	Ribokinase	0.51 ± 0.07	***	7.32
3373:854_CL1Contig1:f	*RGS2*	Regulator of G-protein signaling 2	3.47 ± 0.77	*	3.13
33911:29637_CL1Contig1:r	*SBSN*	Suprabasin	20.37 ± 1.56	***	21.54
TC204140:f	*SDC1*	Syndecan 1	2.55 ± 0.57	*	13.44
2269:10415_CL1Contig1:f	*SDC4*	Syndecan 4	13.79 ± 1.71	***	6.29
TC238171:f	*SERPINE1*	Serpin peptidase inhibitor, clade E, member 1	3.05 ± 0.50	*	14.47
31918:32605_CL1Contig2:f	*SMOX*	Spermine oxidase	33.26 ± 7.94	**	6.29
5977:12455_CL1Contig1:r	*TNFAIP6*	Tumor necrosis factor alpha-induced protein 6	2517 ± 105	***	30.54
19103:6332_CL1Contig1:f	*TNFRSF12A*	Tumor necrosis factor receptor superfamily, member 12A	0.52 ± 0.17	*	3.04
9402:4028_CL1Contig1:r	*UAP1*	UDP-N-acetylglucosamine pyrophosphorylase 1	8.01 ± 1.11	***	5.71
	FSH vs. Control - underexpressed				
707:4250_CL1Contig1:f	*FOS*	Proto-oncogene protein c-fos	8.56 ± 2.43	*	0.15
1893:12937_CL1Contig1:f	*FOXO1*	Forkhead box O1	0.90 ± 0.09	ns.	0.29
6206:5117_CL1Contig1:f	*FST*	Follistatin	0.21 ± 0.02	***	0.10
15012:1275_CL1Contig1:f	*HS3ST1*	Heparan sulfate (glucosamine) 3-O-sulfotransferase 1	0.80 ± 0.12	ns.	0.32
TC210305:f	*IRS1*	Insulin receptor substrate 1	0.98 ± 0.25	ns.	0.14
NM_213880.1	*JUN*	Jun proto-oncogene	1.03 ± 0.14	ns.	0.06
2480:21101_CL1Contig1:f	*PCNA*	Proliferating cell nuclear antigen	1.16 ± 0.12	ns.	0.29
18375:27730_CL1Contig1:r	*PNRC1*	Proline-rich nuclear receptor coactivator 1	0.94 ± 0.15	ns.	0.21
14891:209_CL4Contig1:f	*PPARG*	Peroxisome proliferator-activated receptor gamma	0.45 ± 0.04	***	0.32
1456:37319_CL1Contig1:f	*PTGES*	Prostaglandin E synthase	0.70 ± 0.10	**	0.26
12586:12773_CL1Contig1:f	*RGS3*	Regulator of G-protein signaling 3	0.78 ± 0.10	*	0.17
TC229705:f	*TRIB2*	Tribbles pseudokinase 2	0.84 ± 0.10	ns.	0.33
	AREG/EREG vs. Control - overexpressed				
13062:45360_CL23Contig1:f	*ANXA1*	Annexin A1	0.68 ± 0.11	*	3.68
1117:45360_CL10Contig1:r	*ANXA2*	Annexin A2	2.51 ± 0.41	**	4.41
45:916_CL3Contig1:f	*CTGF* (*CCN2*)	Connective tissue growth factor	2.20 ± 0.25	***	6.19
6532:35657_CL1Contig1:r	*CYP11A1*	Cytochrome P450, family 11, subfamily A, polypeptide 1	2.68 ± 0.44	**	5.82
14983:2713_CL1Contig1:f	*EIF4E*	Eukaryotic translation initiation factor 4E	2.56 ± 0.72	ns.	4.14
977:13547_CL1Contig2:r	*ITPKA*	Inositol-trisphosphate 3-kinase A	5.41 ± 1.22	*	3.36
5795:24780_CL1Contig1:r	*MARCKSL1*	MARCKS-like 1	4.96 ± 0.72	***	3.41
31453:2453_233792:f	*PGR*	Progesterone receptor	4.01 ± 0.58	***	5.25
33911:29637_CL1Contig1:r	*SBSN*	Suprabasin	4.33 ± 0.40	***	4.34
TC204140:f	*SDC1*	Syndecan 1	1.01 ± 0.01	ns.	5.88
TC238171:f	*SERPINE1*	Serpin peptidase inhibitor, clade E, member 1	6.73 ± 0.65	***	33.82
19103:6332_CL1Contig1:f	*TNFRSF12A*	Tumor necrosis factor receptor superfamily, member 12A	4.34 ± 0.53	**	7.70
	AREG/EREG vs. Control - underexpressed				
707:4250_CL1Contig1:f	*FOS*	Proto-oncogene protein c-fos	3.29 ± 0.57	**	0.09
1893:12937_CL1Contig1:f	*FOXO1*	Forkhead box O1	1.06 ± 0.25	ns.	0.33
6206:5117_CL1Contig1:f	*FST*	Follistatin	1.01 ± 0.07	ns.	0.25
9260:5151_CL1Contig1:f	*GADD45A*	Growth arrest and DNA-damage-inducible, alpha	1.20 ± 0.32	ns.	0.35
2134:17017_CL1Contig1:f	*ID1*	Inhibitor of DNA binding 1protein	1.24 ± 0.16	ns.	0.31
NM_213880.1	*JUN*	Jun proto-oncogene	0.54 ± 0.02	***	0.04
30832:5168_40390252:f	*NR5A2*	Nuclear receptor subfamily 5, group A, member 2	1.06 ± 0.08	ns.	0.31
18375:27730_CL1Contig1:r	*PNRC1*	Proline-rich nuclear receptor coactivator 1	0.65 ± 0.16	*	0.32
3373:854_CL1Contig1:f	*RGS2*	Regulator of G-protein signaling 2	1.29 ± 0.09	**	0.34
12586:12773_CL1Contig1:f	*RGS3*	Regulator of G-protein signaling 3	0.36 ± 0.03	***	0.13
TC229705:f	*TRIB2*	Tribbles pseudokinase 2	0.94 ± 0.11	ns.	0.77

*ns*. not significant. *P < 0.05; ** *P* < 0.01; *** *P* < 0.001

**Fig. 6:**
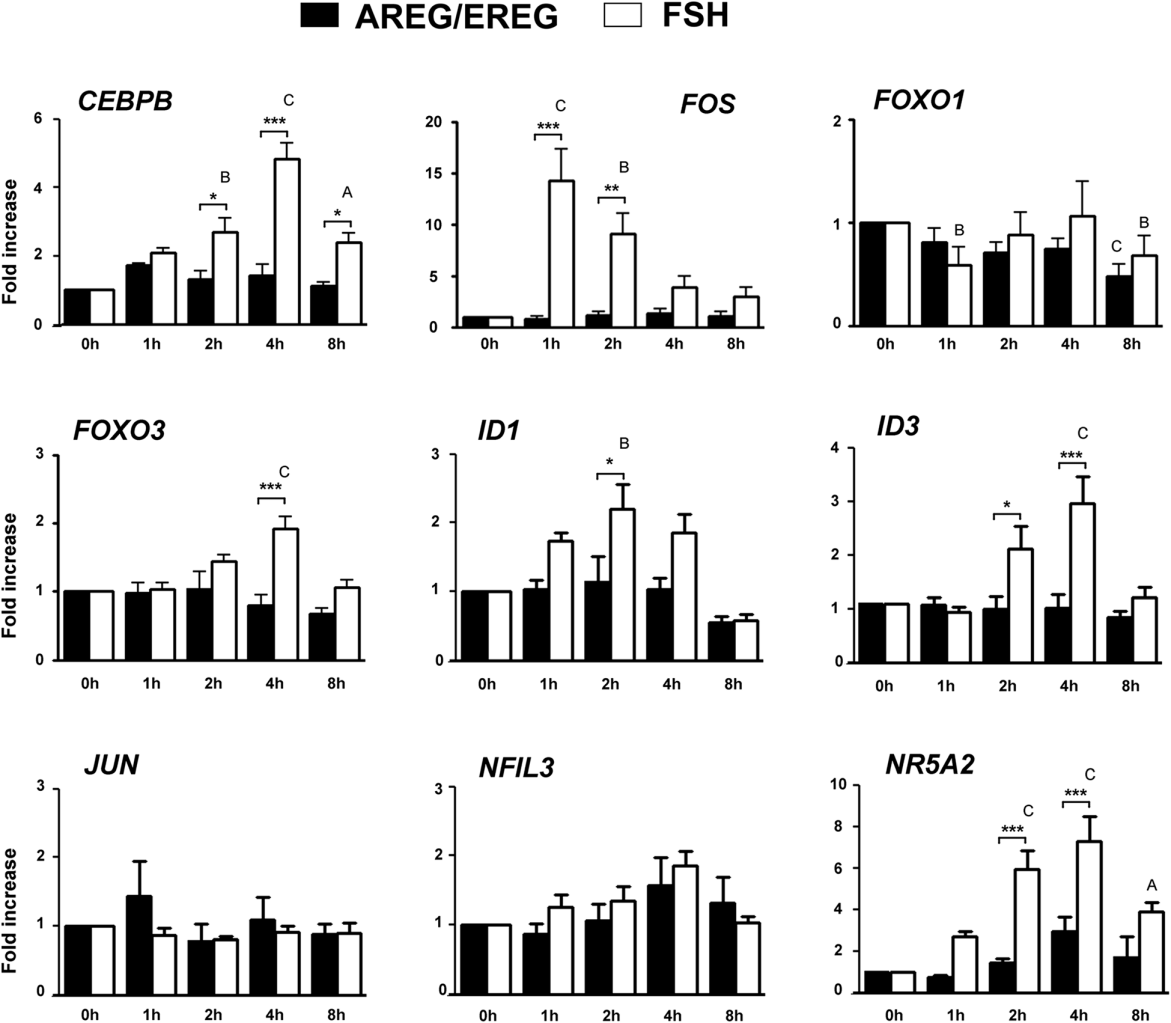
Expression of transcription factors in COCs cultured with AREG/EREG or FSH for various lengths of time. The relative abundance of specific gene mRNA is expressed in arbitrary units as fold increases in the specific gene /*HPRT* ratio over the level found in the control group of COCs at the time 0 h

*Transcription factors*. The results obtained from the AREG/EREG vs. FSH microarray led us to suggest the hypothesis that different expression profiles of AREG/EREG- and FSH-stimulated cells might be caused by alterations in the expression of genes coding the key transcription factors. Therefore, the expression levels of 9 transcription factors were measured by qRT-PCR at various time points during the culture (Fig. 6). In general, FSH was found to affect transcription factor expression more efficiently than AREG/EREG. Figure 6 shows that FSH significantly stimulated the expression of *CEBPB*, *FOS*, *FOXO3*, *ID1*, *ID3* and *NR5A2*. Except for *FOS* and *ID1*, the patterns indicate the accumulation of mRNAs reaching its maximum at 4 and 2 h of culture, respectively. The amount of *FOS* mRNA quickly and dramatically increased within the first hour of culture and then the concentration progressively fell in the subsequent time intervals over the whole culture period. Surprisingly, the expression of *JUN*, a partner transcription factor of *FOS*, was neither affected by FSH- nor by AREG/EREG-stimulation. The expression of *NFIL3* was also unaffected by any type of stimulation.

## Discussion

### Design of the study

We have shown previously that an exposure of COCs to FSH as brief as 3 h, followed by culture in hormone-free medium, is sufficient to initiate the synthesis of hyaluronic acid, cumulus expansion and the resumption of pig oocyte meiosis [34]. The rapid expression of the genes critical for meiotic resumption and cumulus expansion (*HAS2*, *PTGS2*, *TNFAIP6*, *AREG*, and *EREG*) was confirmed in our previous studies [6, 28, 35]; the expression of some regulatory genes inclusive of *AREG* became downregulated by 4 h after FSH addition [28]. With the aim of detecting the primary changes in cumulus cell transcriptomes evoked by FSH or AREG/EREG, we compared non-treated controls and treated groups 3 h after the onset of culture. The data reported in this paper demonstrate that the early alterations in the gene expression profile of cumulus cells do not exclusively pertain to genes involved in the regulation of reproductive functions such as cumulus cell extracellular matrix synthesis and organization, steroidogenesis, ovulation and luteinization, but they cover a broad spectrum of cellular functions. Next, the presented data document that even though both FSH and the EGF-like factors can stimulate events associated with ovulation and final oocyte maturation, essential differences exist in their impact on the modification of the gene expression profile of the cumulus cells.

### ECM formation

We found that FSH increased the expression of a variety of genes involved in extracellular matrix formation and remodeling, such as the cumulus expansion-related genes *HAS2* and *TNFAIP6*. HAS2 is responsible for the formation of hyaluronan, the main component of the expanded cumulus. Additionally, the expression of *HAS2* in human cumulus cells positively correlates with developmental competence of the oocyte [36, 37]. TNFAIP6 is required for the formation of covalent bonds between hyaluronan and the heavy chains of the inter-α-trypsin inhibitor family proteins [38]. Female mice lacking *Tnfaip6* demonstrated severe subfertility and impaired cumulus expansion [39]. Furthermore, FSH promoted the expression of A disintegrin and metalloproteinase with thrombospondin motifs 1 (*ADAMTS1*). In mice, ADAMTS1 is required for the structural remodeling of ovarian follicle and cumulus expansion resulting in ovulation and successful fertilization [40]. In accordance with these findings, *Adamts1* null mice demonstrated impaired female (but not male) fertility caused by morphological abnormalities in the uterus and ovaries [41]. FHS also increased the expression of carbohydrate sulfotransferase 7 (*CHST7*) which may regulate hyaluronan binding to its receptor CD44 by chondroitin sulfonation of CD44 [42].

Both FSH and AREG/EREG enhanced the expression of syndecan 4 (*SDC4*), a heparan sulfate proteoglycan that provides a mechanical link between the extracellular matrix and cytoskeleton [43]. The higher *SDC4* expression in human cumulus cells may predict normal embryo development and pregnancy [44]. Although both culture conditions increased the expression of syndecan 1 (*SDC1*) as assessed by microarray (*SDC1*), qRT-PCR revealed that *SDC1* expression is unaffected by AREG/EREG stimulation. However, the expression of heparan sulfate glucosamine 3-O-sulfotransferase 1 (*HS3ST1*) was increased in COC cultured with FSH, indicating the involvement of heparan sulfate and heparan sulfate-binding proteoglycans in extracellular matrix remodeling or signal transduction in response to gonadotropins or EGF-like peptides. Furthermore, AREG/EREG (but not FSH) enhanced the expression of connective tissue growth factor (*CTGF*), a non-structural matricellular protein that interacts with heparan-sulfate-containing proteoglycans, including syndecans [45]. CTGF has been intensively investigated for its role in the growth and differentiation of granulosa cells and in the formation of the *corpus luteum* [46]. Ovarian and uterine *Ctgf* conditional knockout mice exhibit severe subfertility due to multiple reproductive defects including disrupted follicle development and decreased ovulation rates, despite normal cumulus expansion [47]. Interestingly, the phenotype of *Ctgf* null mice is similar to the phenotype of *Smad4*- or activin-deficient mice, suggesting that CTGF may be a mediator of SMAD4 and activin signaling [47]. In porcine ovaries, the *CTGF* mRNA level of granulosa cells increases as a function of follicular development to a maximum in small antral follicles but *CTGF* mRNA is underexpressed in large antral follicles [48]. These data are in accordance with other studies focused on the role of CTGF in ovarian follicle growth and development [46, 49].

### Transcription factors

Both FSH and AREG/EREG treatment affected the expression levels of various genes involved in transcription regulation. FSH promoted the expression of *CEBPB* and *CEBPD*, the members of a family of basic-leucine zipper transcription factors that interact with the CCAAT box motifs present in gene promoters. CEBPA and CEBPB have been previously found to be essential for ovulation and luteinization in mice [50]. Double-mutant females lacking both *Cebpa* and *Cebpb* genes in granulosa cells failed to ovulate and were completely infertile [50]. Mice null for CEBPB were subfertile and CEBPB seems to play an important role in the AREG-induced expression of *Tnfaip6* and *Ptgs2* in mouse COCs [11]. In pigs, *CEBPB* mRNA significantly increased after ovulation in the *corpus hemorrhagicum* [51]. Furthermore, the CEBPB-dependent transcription of the key genes *Areg*, *Ereg* and *Ptgs2* is inhibited by the interaction of CEBPB with the NFIL3 transcription factor on the gene promoters [52]. Interestingly, *NFIL3* expression was found to be unaffected by culture with AREG/EREG in our study, but it was only increased by FSH.

Quantitative RT-PCR analysis also revealed that FSH, but not AREG/EREG, increased the expression of *NR5A2*, a transcription factor essential for female fertility. *NR5A2* transcripts were highly abundant in granulosa cells and distinctly absent in *theca interna* and ovarian stroma [53]. Conditional knockout of *Nr5a2* in granulosa cells results in infertility, null mice are not able to ovulate due to the failure of preovulatory follicle tissue remodeling caused by abnormally low mRNA levels for proteases implicated in the ovulatory process [10]. NR5A2 seems to be essential for progesterone production [54] and the transcriptional regulation of a variety of genes important for steroidogenesis, such as *Star*, *Cyp11a1*, *Fdx1*, and *Hsd3b1* [55–57]. In accordance with such findings, NR5A2 was found to be required for the formation and maintenance of the *corpus luteum*, promotion of decidualization and for placental formation in mice [57]. However, granulosa-specific targeted disruption of the *Nr5a2* gene yielded contradictory findings about a possible role of this transcription factor in cumulus expansion [10, 54].

Surprisingly, *FOS* was rapidly but transiently induced by FSH within 1 h after addition to culture media. These data are consistent with previously published results in rats [58, 59]. In pigs, FOS is present in preovulatory follicles but lower in the corpus luteum than in antral follicles [60], indicating possible downregulation by LH signaling. Interestingly, the FSH vs. control microarray revealed that BATF3, an inhibitor of the AP1 complex [61, 62], was overexpressed in response to FSH and the transcript was assessed as underexpressed in the AREG/EREG and FSH microarray. However, the role of AP1/FOS during folliculogenesis and ovulation is poorly understood, and it may be involved in many cellular processes, e. g. in the regulation of steroidogenesis [63] or granulosa cell proliferation [64].

In contrast to AREG/EREG, FSH induced the expression of the *ID1* and *ID3* transcription factors. In ovine follicles, the ID1 and ID3 proteins are present in granulosa cells, particularly in the peri-oocytic region [65], indicating the involvement of oocyte-derived factors such as GDF9 and BMP15. Indeed, GFP9 and GMP15 were found to stimulate *ID1* expression in ovine granulosa cells, and both *ID1* and *ID3* mRNA levels were increased by BMP6 and decreased by activin A [65]. In porcine granulosa cells, FSH promoted the expression levels of *ID2* and *ID3* [46]. We also found that AREG/EREG (but not FSH) enhanced the expression of Cbp/P300-interacting transactivator 2 (*CITED2*). This transcription factor is involved in gonad development [66] and negatively regulates the expression of genes coding matrix metalloproteinases in chondrocytes [67].

In agreement with the microarray results, qRT-PCR confirmed the downregulation of *PPARG* mRNA in COCs cultured in the presence of FSH. In mice, the level of *Pparg* mRNA is enhanced in a progesterone-dependent manner in the granulosa cells of PMSG-primed mice treated with hCG [68]. Granulosa cell-specific deletion of *Pparg* resulted in a significant impairment of follicle rupture and subfertility [68]. However, other *Pparg* null mice didn’t exhibit any problems in their ovulation process and were subfertile due to abnormal implantation [69].

### Polyamine metabolism

FSH also stimulated the expression of *ODC1* and *SMOX*, enzymes involved in polyamine metabolism. Xenopus oocytes lacking ODC1 activity exhibit high levels of reactive oxygen species and high caspase 3 activity [70]. Female mice overexpressing *Sat1 mRNA*, whose product is a rate-limiting enzyme in polyamine catabolism, were found to be infertile due to ovarian hypofunction and hypoplastic uteri [71]. The ovaries of these mice contained the primary and small secondary follicles, but larger developing follicles and *corpora lutea* were absent, indicating disruption of the late phases of folliculogenesis and ovulation. SMOX oxidizes spermine to produce spermidine, 3-aminopropanal and hydrogen peroxide [72]. Exogenous hydrogen peroxide on its own effectively induced cumulus expansion in mouse COCs [73]. Moreover, broad-range scavengers of reactive oxygen species (ROS) inhibited LH-induced cumulus expansion, activation of EGFR and MAPK3/1 and expression of cumulus expansion-related genes (*Has2*, *Ptgs2*, *Tnfaip6*) [73]. Antioxidants may even reduce the ovulation rate in mice [73, 74] and rabbits [75]. Interestingly, AREG/EREG induced the expression of neutrophil cytosol factor 2 (*NCF2*) which is required for the activation of NADPH oxidase and superoxide production [76].

### ROS

The generation of ROS in rat follicles induced by LH was previously described to be coupled with mitochondrial steroidogenesis via Star/Cyp11A1 [77]. To protect against oxidative damage and subsequent apoptosis, the increase in steroidogenic capacity is correlated with increased activity of the antioxidants [78]. Both FSH and AREG/EREG promoted expression of glutaredoxin 1 (*GLRX*), which reduces low-molecular-weight disulfides and proteins [79]. Moreover GLRX was shown to reduce mixed disulfides between various signaling proteins and glutathione, regenerating their activities and affecting signal transduction and cellular response [79]. FSH induced the expression of a variety of genes involved in the response to oxidative damage (*EPHX3*, *GSTM3*, *KLHDC10*, *PRDX2*, *MT2A*, and *MT3*). Glutathione S-transferase M3 (GSTM3) has well-established roles in the detoxification and clearance of a variety of electrophilic compounds, including xenobiotics as well as substances generated by ROS damage to intracellular molecules [80], but also acts as a tumor suppressor [81]. Peroxiredoxin 2 (PRDX2) is an antioxidant enzyme reducing hydrogen peroxide and alkyl hydroperoxides by using reducing agents such as thioredoxin [82]. *Prdx2* was previously found to be expressed in the ovaries of eCG-primed rats in response to hCG administration, localized in the granulosa and theca cells of preovulatory follicles in the ovaries of eCG-primed rats [74]. PRDX2 is proposed to be part of a control mechanism for the balance between the production and elimination of hydrogen peroxide during the periovulatory period [74]. In mouse granulosa cells, suppression of the *Prdx2* gene using siRNA inhibits cell proliferation, promotes apoptosis and increases the production of endogenous hydrogen peroxide [83]. Furthermore, the expression of *PRDX2* in the cumulus cells of women positively correlates with embryo quality [84].

### Coagulation factors

Surprisingly, both FSH and AREG/EREG induced the expression of genes involved in “blood coagulation”, namely plasminogen activator inhibitor 1 (*SERPINE1*), tissue factor (*F3*), and tissue factor pathway inhibitor 2 (*TFPI2*). Moreover, FSH itself increased the expression levels of urokinase plasminogen activator surface receptor (*PLAUR*), tissue-type plasminogen activator (*PLAT*), junctional adhesion molecule A (*F11R*), CD9 antigen (*CD9*). PLAT, SERPINE1 and PLAUR are components of the plasminogen activation system, which regulates the conversion of plasminogen to plasmin. Numerous studies have reported the expression of these genes in antral follicles after exposure to a gonadotropin surge [85–87], but the role of plasmin during the ovulation process is still unclear. Plasmin is assumed to play a role in collagen breakdown and stigma development [88]. On the other hand, plasminogen-deficient females exhibited normal ovulation efficiency compared to plasminogen wild-type mice [89] and the reduced fertility of plasminogen knockout females may be caused by poor general health [90]. Some studies indicate that plasmin activity is involved in cumulus expansion and/or extracellular matrix remodeling [91], and progesterone production by the *corpus luteum* [92]. In pigs, plasmin detached sperm bound to *zona pellucida* and thus reduced the risk of polyspermy [93].

## Conclusions

The stimulation of in vitro cultured COCs by both FSH and EGF-like factors caused a rapid and extensive rearrangement of gene expression patterns in cumulus cells. These early changes in the cumulus cell transcriptome not only pertained to events associated with the final maturation of oocytes and expansion of the cumulus cells, but they covered a broad spectrum of general cellular functions including signal transduction, regulation of the cell cycle, growth and proliferation, cytoskeleton and microtubule organization, metabolism, apoptosis, response to stress and last but not least the regulation of transcription. As expected, the impact of FSH on cumulus cell gene transcription was higher than the impact of EGF-like factors in terms of the number of cell functions affected as well as the number of over- and underexpressed genes. As for the regulation of ovarian functions, both FSH and EGF-like factors overexpressed genes involved in the post-ovulatory switch in steroidogenesis (*CYP11A1* and *PGR*) and tissue remodeling (*F3*, *SERPINE1*, and *TIMP1*). However, FSH was remarkably more efficient in the up-regulation of several specific genes that are considered to be essential for the successful ovulation of matured oocytes (*ADAMTS1*, *HAS2*, *HSD17B7*, *PLAT*, *PLAUR*, and *TNFAIP6*) and that have also been reported to play an essential role in the maturation of cumulus-enclosed oocytes in vitro (*ADAMTS1*, *HAS2*, and *TNFAIP6*).

We have shown in our previous work that EGF-like peptides do not mimic all effects of FSH on cultured pig COCs, nevertheless they provided oocytes with the developmental competence comparable or even higher than those stimulated with gonadotropins [28]. These data were confirmed in further studies with pig and bovine COCs [32, 33, 94]. Moreover, a synergistic effect of AREG and oocyte-derived paracrine factors (GDF9, BMP15) and cAMP on promotion of developmental competence of in vitro cultured oocytes was recently reported [32, 33]. The molecular background of this feature was identified as a promotion of EGFR signaling system in the somatic cell compartment, which was critical for successful maturation of less competent COCs isolated from small size follicles [33]. Taken together with the data of the present study, we may conclude that in a simplified model of in vitro culture of COCs isolated from growing follicles, both cAMP-PKA signaling induced by gonadotropins and action of oocyte-derived paracrine signaling are required for proper function of the EGFR activated by EGF-like peptides. The application of this knowledge may increase efficiency of the current IVM/IVF systems for use in animal breeding, biomedicine and for the treatment of infertility.

## Additional files

Additional file 1: Table S1. List of primers used for microarray data analysis verification. List and data of primers used in qRT-PCR. F, forward primer; R, reverse primer. (XLSX 18 kb) Additional file 2: Table S2. List of over- and underexpressed genes in cumulus cells stimulated with FSH or AREG/EREG depicted in Venn diagrams. A complete list of genes depicted in Venn diagrams (Fig. 2). (XLSX 47 kb)

## Abbreviations

ADAMTS1: A disintegrin and metalloproteinase with trombospondin motif 1
ADM: Adrenomedullin
AKT: v-akt murine thymoma viral oncogene homolog
ANXA1: Annexin A1
ANXA2: Annexin A2
ANXA5: Annexin A5
AP1: activator protein 1
AREG: Amphiregulin
BATF3: Basic leucine zipper transcriptional factor ATF-like 3
BMP6: Bone morphogenetic protein 6
BMP15: Bone morphogenetic protein 15
BMPR1B: Bone morphogenetic protein receptor type-1B
BTC: Betacellulin
CCL4L1: Chemokine (C-C motif) ligand 4-like 1
CEBPA: CCAAT/enhancer binding protein alpha
CEBPB: CCAAT/enhancer binding protein beta
CD9: CD 9 antigen
CEBPD: CCAAT/enhancer binding protein delta
CHST7: Carbohydrate sulfotransferase 7
CITED2: Cbp/P300-interacting transactivator 2
COC: Cumulus-oocyte complex
CREB: cAMP response element binding protein
CREM: cAMP response element modulator
CRYAB: Crystallin, alpha- B
CTSL: Cathepsin L
CTGF: Connective tissue growth factor
CYP11A1: Cytochrome P450, family 11, subfamily A, polypeptide 1
EGF: Epidermal growth factor
EGFR: Epidermal growth factor receptor
EPHX3: Epoxide hydrolase 3
EREG: Epiregulin
F3: Coagulation factor 3
F11R: F11 receptor
FBLN5: Fibulin-5
FDX1: Ferredoxin 1
FGF9: Fibroblast growth factor 9
FOS: Proto-oncogene protein c-fos
FOXO1: Forkhead box protein O1
FOXO3: Forkhead box protein O1
FST: Follistatin
FSH: Follicle stimulating hormone
GCLC: Glutamate cysteine ligase catalytic subunit
GDF9: Growth differentiation factor 9
GLRX: Glutaredoxin 1
GRB14: Growth factor receptor-bound protein 14
GRB14: Growth factor receptor-bound protein 14
GSTM3: Glutathione S-transferase Mu 3
GV: Germinal vesicle
GVBD: Germinal vesicle breakdown
HA: hyaluronic acid
HAS2: Hyaluronan synthase 2
HS3ST1: heparan sulfate glucosamine 3-O-sulfotransferase 1
HSD17B7: Hydoxysteroid (17-beta) dehydrogenase 7
HSD3B1: Hydroxy-delta-5-steroid dehydrogenase, 3 beta- and steroid delta-isomerase 1
ID1/3: Inhibitor of DNA-binding protein 1/3
IRS1: Insulin receptor substrate 1
JUN: Jun proto-oncogene
KLHDC10: Kelch domain-containing protein 10
LH: Luteinizing hormone
LTA4H: Leukotriene A-4 hydrolase
MII: metaphase II
MAPK3/1: Mitogen-activated protein kinase 3/1
MT2A: Metallothionein 2A
MT3: Metallothionein 3
NCF2: Neutrophil cytosol factor 2
NFIL3: Nuclear factor interleukin-3-regulated
NR4A1: Nuclear receptor subfamily 4, group A, member 1
NR5A2: Nuclear receptor subfamily 5, group A, member 2
NRIP1: Nuclear receptor-interacting protein 1
ODC1: Ornithine decarboxylase 1
OLR1: Oxidized low-density lipoprotein receptor 1
PGR: progesterone receptor
PI3K: phosphoinositide-3-kinase
PKA: Protein kinase A
PLAT: plasminogen activator, tissue
PLAUR: Plasminogen activator, urokinase receptor
PNRC1: Proline-rich nuclear receptor coactivator 1
PPARG: Peroxisome proliferator-activated receptor gamma
PRDX2: Peroxiredoxin-2
PTGS2: Prostaglandin-endoperoxide synthase 2
PTGER: Prostaglandin E receptor 2
PTGES: Prostaglandin E synthase
RGS3: Regulator of G-protein signaling 3
SAT1: Spermidine/spermine N1-acetyltransferase 1
SBSN: Suprabasin
SDC1: Syndecan 1
SDC4: Syndecan 4
SERPINE1: Serpin peptidase inhibitor, clade E, member 1
SMAD2: SMAD family member 2
SMAD4: SMAD family member 4
SMOX: Spermine oxidase
SNAP: S-nitroso-N-acetyl-penicillamine
TAC1: Tachykinin precursor 1
TFPI2: Tissue factor pathway inhibitor 2
TGFB1: Transforming growth factor beta-1
TNFAIP6: Tumor necrosis factor alpha-induced protein 6
TXNIP: Thioredoxin-interacting protein
